# xCT-Driven Expression of GPX4 Determines Sensitivity of Breast Cancer Cells to Ferroptosis Inducers

**DOI:** 10.3390/antiox10020317

**Published:** 2021-02-20

**Authors:** Namgyu Lee, Anne E. Carlisle, Austin Peppers, Sung Jin Park, Mihir B. Doshi, Meghan E. Spears, Dohoon Kim

**Affiliations:** 1 Cell and Cancer Biology, Department of Molecular, University of Massachusetts Medical School, Worcester, MA 01604, USA; namgyu.lee@umassmed.edu (N.L.); anne.carlisle@umassmed.edu (A.E.C.); austin.peppers@umassmed.edu (A.P.); mihir.doshi@umassmed.edu (M.B.D.); meghan.spears@umassmed.edu (M.E.S.); 2 Program in Molecular Medicine, University of Massachusetts Medical School, Worcester, MA 01604, USA; sungjin.park@umassmed.edu

**Keywords:** ferroptosis, GPX4, Erastin, Rsl-3, breast cancer, selenium, lipid peroxidation

## Abstract

Inducers of ferroptosis such as the glutathione depleting agent Erastin and the GPX4 inhibitor Rsl-3 are being actively explored as potential therapeutics in various cancers, but the factors that determine their sensitivity are poorly understood. Here, we show that expression levels of both subunits of the cystine/glutamate antiporter xCT determine the expression of GPX4 in breast cancer, and that upregulation of the xCT/selenocysteine biosynthesis/GPX4 production axis paradoxically renders the cancer cells more sensitive to certain types of ferroptotic stimuli. We find that GPX4 is strongly upregulated in a subset of breast cancer tissues compared to matched normal samples, and that this is tightly correlated with the increased expression of the xCT subunits SLC7A11 and SLC3A2. Erastin depletes levels of the antioxidant selenoproteins GPX4 and GPX1 in breast cancer cells by inhibiting xCT-dependent extracellular reduction which is required for selenium uptake and selenocysteine biosynthesis. Unexpectedly, while breast cancer cells are resistant compared to nontransformed cells against oxidative stress inducing drugs, at the same time they are hypersensitive to lipid peroxidation and ferroptosis induced by Erastin or Rsl-3, indicating that they are ‘addicted’ to the xCT/GPX4 axis. Our findings provide a strategic basis for targeting the anti-ferroptotic machinery of breast cancer cells depending on their xCT status, which can be further explored.

## 1. Introduction

Ferroptosis is a form of cell death that involves an iron-dependent accumulation of lipid peroxides. Several cellular components have emerged as playing key roles in the regulation of ferroptosis. The cystine-glutamate antiporter xCT which is composed of the subunits SLC7A11 and SLC3A2, plays a protective role against ferroptosis by allowing the import of cystine, which is a rate-limiting step in the biosynthesis of the antioxidant molecule glutathione [1]. The enzyme glutathione peroxidase 4 (GPX4) utilizes glutathione as a substrate for reduction, and the activity of GPX4 in reducing lipid peroxidation has been shown to be a key step in the prevention of ferroptosis [2].

GPX4 is a selenoprotein, with a selenocysteine residue in its active site, and thus it requires selenocysteine biosynthesis for its expression and activity [3,4]. The selenocysteine biosynthesis pathway incorporates selenium, such as in the dietary form selenite, to produce selenocysteinyl-tRNA. The antioxidant function of selenium as a nutrient is mediated through selenoproteins such glutathione peroxidases and thioredoxins, including GPX4, thus implicating selenium in ferroptosis as well [5,6]. Furthermore, it was found that elevated SLC7A11 expression in cancer cells drives selenocysteine biosynthesis by promoting selenite reduction and uptake as an early step in selenocysteine biosynthesis [4]. Thus, SLC7A11 allows both the production of glutathione, and the production of the enzyme (GPX4) that utilizes it.

Breast cancer, along with lung cancer, is the most common cancer in the world [7]. While successful treatment paradigms have emerged for breast cancers expressing estrogen, progesterone, or Her2 receptors, triple negative breast cancers (TNBCs) that do not express any of these receptors are refractory to conventional chemotherapeutics and have a markedly poor prognosis [8]. Well-established chemical inducers of ferroptosis, such as the GPX4 inhibitor Rsl-3 and the xCT inhibitor Erastin, and their derivatives, are currently being explored as potential therapeutics for breast cancer therapy and have been shown to exert toxicity in these and other cancer cells [9]. On the other hand, what determines the sensitivity and resistance of cells to these compounds is a critical issue that must be further explored to develop ferroptotic induction as a valid therapeutic strategy for cancer. 

Here, we examined the expression and functional importance of ferroptotic machinery between transformed and nontransformed cells. We observe that expression of the xCT components SLC7A11 and SLC3A2 are elevated in a subset of patient breast tumor tissues compared to normal breast tissue, and that their coordinated expression tightly correlates with GPX4 expression. We find that the xCT inhibitor Erastin disrupts expression of antioxidant selenoproteins by impairing xCT mediated extracellular reduction, which is required for uptake of selenite and selenocysteine biosynthesis, indicating that modulation of selenoprotein expression is a potentially important and previously unrecognized function of Erastin. In a panel of breast cancer and nontransformed immortalized breast epithelial cell lines, we find the breast cancer lines to be resistant to hydrogen peroxide, consistent with increased GPX4 function. Paradoxically, the breast lines are at the same time hypersensitive to the Erastin and Rsl-3 indicating that breast cancer cells are addicted to the xCT/GPX4 axis to prevent ferroptosis. Our findings indicate a therapeutic window and mechanism for compounds targeting the antiferroptotic machinery of breast cancer cells.

## 2. Materials and Methods

All reagents used in this paper are listed in Supplementary Table S1.

### 2.1. Cell Lines and Cell Culture

All cell lines were cultured in a humidified incubator at 37 °C under 5% CO_2_. The cell line information used in this paper is provided in Supplementary Table S2.

### 2.2. Western Blot Analysis

Cells were washed with cold PBS and lysed in RIPA lysis buffer with protease inhibitor (Sigma Aldrich, St. Louis, MO, USA). After 20 min incubation on ice, lysates were centrifuged at 12,000× *g* at 4 °C for 10 min to collect supernatant. Protein concentration of each samples was determined by Bradford assay. Typically, 15–25 μg of total protein were denatured in 6× Lamelli buffer (Boston bioproducts, Boston, MA, USA), loaded per lane on SDS-PAGE gel (Biorad, Hercules, CA, USA), and analyzed by standard immunoblotting. The target proteins were detected by ECL, Pico or diluted Femto substrate (Thermo Fisher Scientific, Waltham, MA, USA). 

### 2.3. Cell Viability Assay

The effect of GPX4 inhibition is affected by cell confluency (31341276). Thus, all viability assays were performed with consistently regarding cell seeding number and time points. For testing toxicity of hydrogen peroxide, Erastin and Rsl-3 in cell lines, 1500 cells were plated in a 96 well plate. Next day, cells were treated with the drugs. Cell viability was measured by CellTiter-Glo Luminescent Assay (Promega, Madison, WI, USA). 

### 2.4. Conditioned Media Thiol Quantification (Ellman’s Test)

10,000 cells were plated per well of a 96 well plate. The next day, the media was changed to 100 μL fresh media containing vehicle or Erastin. After 12 h conditioning, the conditioned media were collected and directly mixed with 50 μL of 10 mM DTNB (5,5′-dithiobis-(2-nitrobenzoic acid)) dissolved in DMSO in another 96 well plate. Absorbance at 450 nm was measured spectrophotometrically in 3 min (DTX880, Beckman Coulter, Indianapolis, IN, USA). The blank value was subtracted as noise, and all values were normalized to that of unconditioned media. The leftover cells in the 96 well plate were subjected to the CellTier-Glo Luminescent Assay after adding fresh media. As phenol red mask the changed color of DTNB, phenol red-free media was used for this assay.

### 2.5. Total Selenium Measurement by ICP-MS Analysis

3 million CAL120 and 4 million MDAMB231 cells were plated in 150 pi dish, and media were changed with that containing vehicle, 12 μM selenite, or 12 μM Erastin the next day. After 2 h of treatment, cells were harvested and washed three times with cold PBS, and weighed. The cell pellets were treated with 500 μM 1:1 H_2_O_2_/HNO_3_ and stood in a safety hood for 12 h at room temperature with periodic venting. Samples were then sonicated for 1 h at 35 kHz and 40 °C with periodic venting. Contents of the Eppendorf tubes were then transferred to a glass digestion tube with ASTM type I water (2 × 1 mL). Tubes were sealed, heated at 140 °C for 2 h, then cooled and diluted to 5 mL with ASTM type I water for selenium analysis.

Selenium measurements were carried out with an Agilent 7500A ICP-MS system which had a standard concentric nebulizer, a Peltier-cooled and double-pass Scott-type spray chamber, torch shield, and standard Nickel interface cones. Between each analytical sample, the probe sample introduction system was rinsed with 10% nitric acid for 60 s to prevent carryover. Calibration curves were made with standard solutions of Se (Ultra Scientific, Kingstown, RI, USA) and QC samples using a multi-element standard (Environmental Express). 

### 2.6. Detection of Lipid Peroxidation

Cells were collected by trypsinization and washed with HBSS. The cell pellets were resuspended with 150 μL of 5 μM BODIPY™ 581/591 C11 lipid peroxidation sensor (Thermo Fisher Scientific, Waltham, MA, USA) in HBSS and incubated at 37 °C. After 20 min incubation, 500 μL of HBSS was added to the stained cells and subjected to FACS analysis with BD LSR II flow cytometer immediately. Briefly, SSC-A and FSC-A gating strategy was applied to remove the cell debris. FSC-H and FSC-A subgating was performed to identify single cell population. Around 10,000 cells gated as single were used for analysis. BD FACS Diva program and FLOWJO10 were used for data collection and data analysis, respectively.

### 2.7. RNA Expression and Prognostic Value SLC7A11 and SLC3A2 in Normal and Tumor Tissues 

Analyses of gene expression data in breast normal and cancer tissues, and disease free survival and overall survival in patients with breast cancer were performed using the web tool GEPIA [10]. Total numbers of samples for each analysis are not designated by the user; instead the GEPIA web tool designates the high and low from the median and provides the total numbers. Total numbers may differ between the different genes (SLC7A11 vs. SLC3A2) due to some samples not meeting the GEPIA criteria for either high or low designation for the given gene.

### 2.8. Processing of Human Breast Tissues

Human breast cancer samples and normal breast tissues were obtained with informed consent from the University of Massachusetts Medical School Biorepository and Tissue Bank using procedures which were conducted under an Institutional Review Board (IRB)-approved protocol. After surgical removal, fresh tumor tissues or normal tissues were immediately snap-frozen in liquid nitrogen and stored at −80 °C. Later, tissues were homogenized in RIPA buffer with complete protease inhibitor cocktail, then centrifuged at 13,000× *g* at 4 °C for 10 min. Samples were normalized for protein content and western blots were performed. Protein bands were quantified using Image J program. Scanned films were inverted, and the intensity of each band was measured, and then the background value was subtracted. The intensity value of protein bands for each sample was normalized to the intensity value for Actin or GAPDH proteins.

### 2.9. Quantification and Statistical Analysis

Results of the viability assay, comparison of protein expressions between normal and cancer tissues, Ellman’s test and total selenium measurement were analyzed using Student’s t test. Disease free survival and overall survival in patients with breast cancer were compared between xCT high and low groups using the Kaplan–Meier method, and significant differences in curves were assessed using the log-rank test. Values of *p* < 0.05 were considered statistically significant, and data marked with a one (*), two (**) or three (***) asterisks indicate *p* values of <0.05, <0.01 and <0.001, respectively.

## 3. Results

### 3.1. Expression of xCT, an Initial Step of Selenocysteine Synthesis Pathway, Is Highly Upregulated in Breast Cancer Tissues and Expression of GPX4 Is High in xCT Positivie Tumors

**Figure 1:**
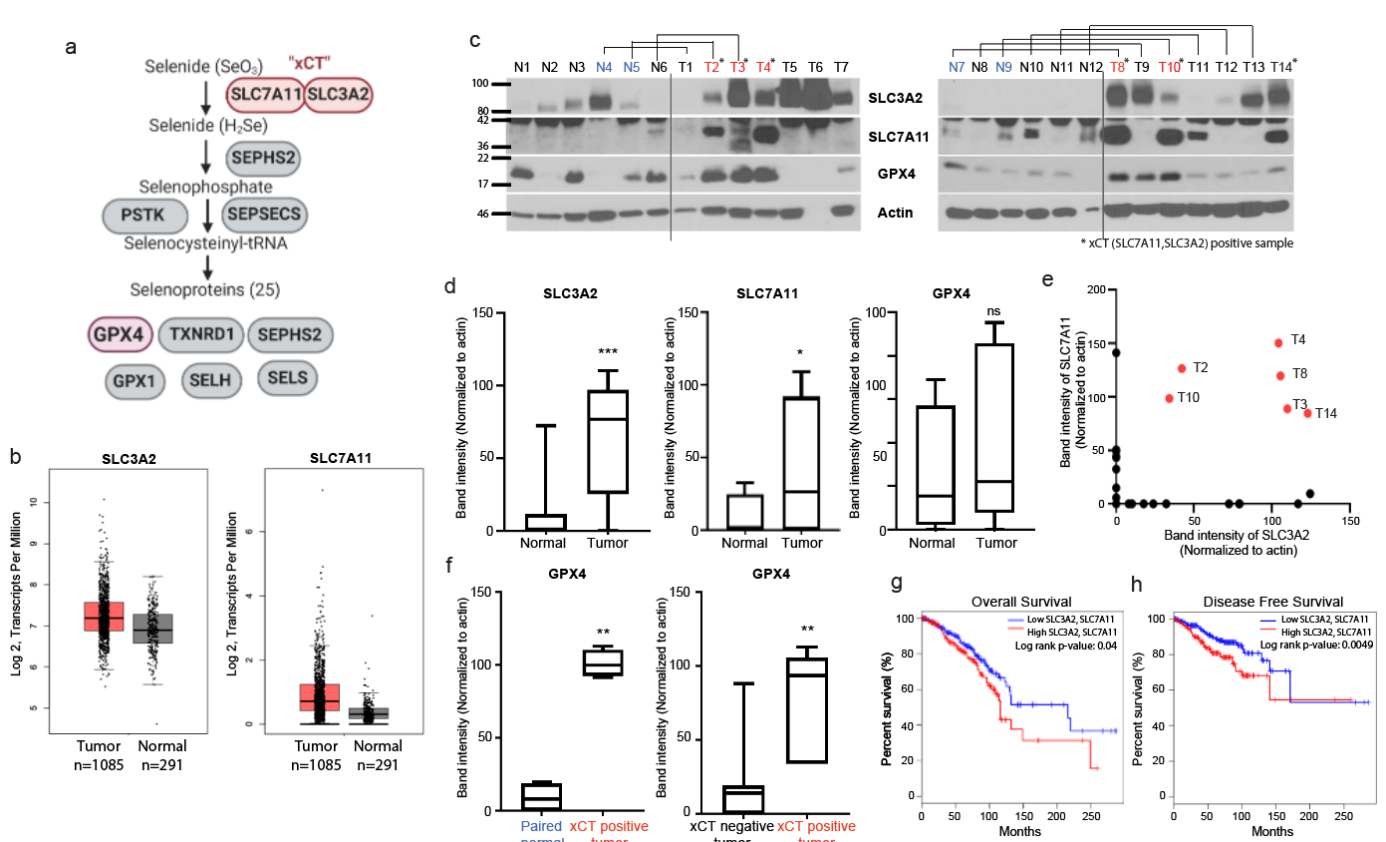
Expression of both subunits of xCT correlates with elevated GPX4 expression in breast cancer patient tumors. (**a**) Schematic diagram of the selenocysteine synthesis pathway which produces selenocysteinyl tRNA required in selenoproteins such as GPX4. Created with Biorender (https://app.biorender.com/). (**b**) Analyses of normalized mRNA transcript levels from tumor (TCGA) and normal breast tissue (GTEx) datasets, conducted using GEPIA (http://gepia2.cancer-pku.cn/) web tool. (**c**) Immunoblots of SLC3A2, SLC7A11, GPX4, and Actin in breast normal and cancer tissues. (**d**) Comparison of Actin-normalized band intensities of SLC3A2, SLC7A11, and GPX4 proteins between all breast normal and cancer tissues. (**e**) Dot plot of Actin-normalized band intensities of SLC3A2 and SLC7A11 in all samples shown in c. Sample defined as xCT positive tumor is indicated with asterisks. (**f**) Comparison of band intensities of GPX4 between xCT positive tumors and their paired normal tissues. The cases of xCT positive tumor and each paired normal tissue are indicated with red and blue in panel (**c**). (**g**,**h**) Expression of both SLC3A2 and SLC7A11 estimates overall survival (**g**) and disease-free survival (**h**) for patients with breast cancer (Median cutoff; Low SLC3A2 and SLC7A11, *n* = 400; High SLC3A2 and SLC7A11, *n* = 400). Data marked with a one (*), two (**) or three (***) asterisks indicate *p* values of <0.05, <0.01 and <0.001. ns: no significance.

As xCT activity was shown to drive the expression of the selenoprotein GPX4 via the selenocysteine biosynthesis pathway (Figure 1a, [4]), we examined the levels of SLC7A11, SLC3A2, and GPX4 in breast normal and cancer tissues to confirm if this pathway is altered in cancer tissues and if GPX4 expression is associated with the expression of xCT. First, we analyzed normalized mRNA transcript levels of SLC7A11 and SLC3A2 from data from The Cancer Genome Atlas (TCGA) compared with normal tissues from (GTEx), using the web tool GEPIA [10], which demonstrated a trend towards increased mRNA levels which was not significant (Figure 1b); similar non-significant trends for increase in both subunits was observed in data mining across multiple cancer types (Supplementary Figure S1a). When directly examining protein levels from patient-derived breast tumor samples and normal breast tissues, we found that the expression levels of the xCT subunits, SLC7A11 and SLC3A2, were significantly upregulated in breast tumor tissues compared with normal tissues (Figure 1c,d). xCT requires both these subunits [4], thus we tried to distinguish between tissues that express only one or both of the subunits. Interestingly, there were 6 cancer tissues out of 14 that expressed significant levels of both SLC7A11 and SLC3A2, which we designated xCT positive tissue (Figure 1e), while none of the normal breast tissues was xCT positive (Figure 1c). Interestingly, when comparing expression across all tumor samples versus all normal tissue samples, in contrast to SLC3A2 and SLC7A11, GPX4 was not significantly overexpressed in cancer tissues compared to normal tissues across the set (Figure 1d). In contrast, when directly comparing the protein expression levels of GPX4 in xCT-positive tumors with their paired (i.e., from the same patient) normal breast tissue, or comparing all xCT positive tumors tissues with all xCT-negative tumor tissues, we saw statistically significant increases of GPX4 expression in the xCT positive group (Figure 1f). Furthermore, analyses of TCGA data indicated that high expression of both subunits of xCT was significantly associated with poor overall survival (Figure 1g) and disease-free survival (Figure 1h) in patients with breast cancer. In contrast, expression of either SLC7A11 or SLC3A2 alone did not have significant prognostic value (Supplementary Figure S2a,b). These findings support the notion that xCT drives the expression of GPX4 [4], and that the elevated expression of both SLC7A11 and SLC3A2 is a determinant for increased xCT function as indicated by GPX4 expression—And is associated with poor prognosis in breast cancer.

### 3.2. Erastin Targets the Selenium Uptake and Selenoprotein Expression Promoting Activity of xCT

**Figure 2:**
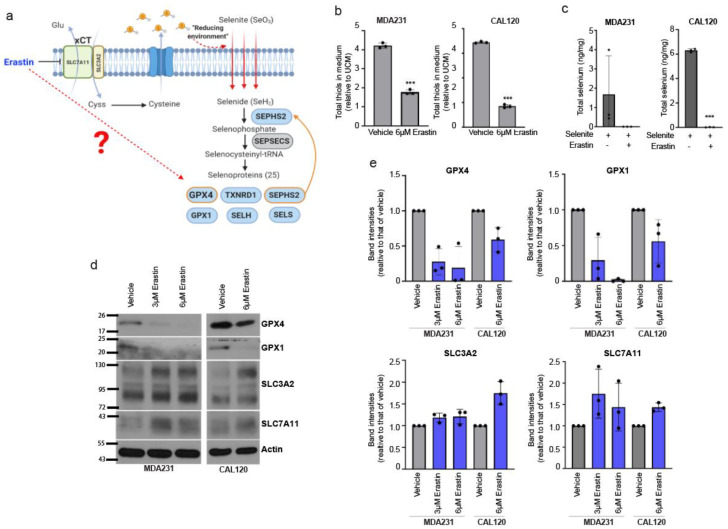
Erastin downregulates the expression of GPX4 and GPX1 via inhibition of selenium uptake. (**a**) Schematic diagram of the proposed role of xCT in the selenoprotein synthesis pathway. Created with Biorender (https://app.biorender.com/). (**b**) Total thiol quantification of conditioned media from vehicle or 6 µM Erastin treated MDAMB231 and CAL120 cells, conditioned for 12 h. Each value was normalized to that of unconditioned medium (UCM), set to 1. (**c**) Selenium uptake in vehicle or Erastin-treated CAL120 and MDAMB231 cells. Total intracellular selenium levels were measured following treatment with 12 µM selenite and/or Erastin for 2 h. (**d**) Immunoblots of GPX1, GPX4, SLC3A2, SLC7A11, and Actin in 3 µM, 6 µM Erastin or vehicle-treated cells for 48 h. (**e**) Quantification of actin-normalized band intensities of GPX4, GPX1, SLC3A2 and SLC7A11 relative to the vehicle, from multiple experiments. Error bars represent the mean ± s.d. (*n* = 3). Three independent experiments were performed for b and c and each experiment contains three biological replicates. The bars for b represent the mean ± s.d. of averaged values from each experiment. The bars for c are the mean ± s.d. of biological triplicates from a representative set. Data marked with a three (***) asterisks indicate *p* values of <0.001.

Erastin is an established xCT inhibitor and inducer of ferroptosis, and is currently being explored as a potential breast cancer therapeutic agent. The ferroptosis inducing activity of Erastin and other xCT inhibitor compounds have been primarily attributed to the inhibition of cystine import leading to glutathione depletion, as cysteine which can be formed from the reduction of cystine is a rate-limiting precursor for glutathione biosynthesis [11]. The role of xCT in promoting selenium uptake and selenoprotein expression suggested to us that Erastin may also function to disrupt the selenium-dependent expression of GPX4, which given the role of GPX4 in ferroptosis may greatly affect a cancer cell’s sensitivity or resistance to ferroptosis. xCT is thought to promote selenium entry into a cell because some of the imported cystine is intracellularly reduced to cysteine and exported, providing extracellular reduced thiol groups which allow selenite (SeO_3_) to be reduced to selenide to initiate the selenocysteine biosynthesis pathway (Figure 2a) [4,12]. We found that Erastin treatment in breast cancer cells diminished the levels of extracellular thiols (Figure 2b), eliminated their selenite uptake (Figure 2c), and reduced expression of the selenoprotein antioxidants GPX1 and GPX4 (Figure 2d,e), indicating that Erastin is disrupting xCT-mediated extracellular reduction which leads to selenium uptake and selenoprotein production in these cells. These findings support the model for xCT function in GPX4 expression which we previously proposed [4]: that xCT, by allowing cystine import which is in turn reduced and exported to provide extracellular thiols, results in selenite reduction and uptake, leading to production of selenoproteins such as GPX4. Erastin, by inhibiting the cystine import function of xCT, hinders this process and the production of GPX4. These doses of Erastin (3 or 6 μM) did not cause significant toxicity at the time point for these experiments (Supplementary Figure S3a,b), indicating that cellular toxicity is not a reason for reduced extracellular thiols, selenium uptake, and GPX expression. Furthermore, we observed that Erastin did not decrease, and actually slightly increased, expression of SLC3A2 and SLC7A11 subunits in these cells (Figure 2d,e), supporting that Erastin worked through direct inhibition of xCT activity rather than impairing xCT subunit expression. Collectively, these findings further support the role of xCT in driving the expression of antiferroptotic agent GPX4, and suggest that inhibition of the selenocysteine biosynthesis axis is an important consequence of xCT inhibition by Erastin.

### 3.3. Breast Cancer Cells Have Increased Resistance against Cell Death Induced by Reactive Oxygen Species Which Correlates with xCT Expression

**Figure 3:**
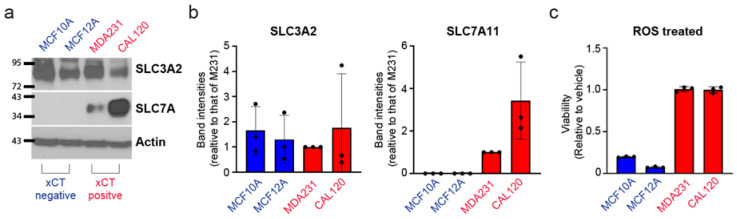
TNBC lines MDAMB231 and CAL120 are xCT-positive and have increased resistance against hydrogen peroxide compared to nontransformed, xCT-negative lines MCF10A and MCF12A. (**a**) Immunoblots of the xCT subunits SLC3A2 and SLC7A11 in TNBC cell lines (labeled red) and nontransformed lines (labeled blue). MCF10A and MCF12A nontransformed lines are categorized as xCT negative, as they express SLC3A2 but not the SLC7A11 subunits. (**b**) Quantification of actin normalized band intensities of SLC3A2 and SLC7A11, relative to vehicle, from multiple experiments. (**c**) Cell viability of each of these cells upon treatment with 200 μM hydrogen peroxide for 24 h, measured using CellTiterGlo, and expressed relative to each line treated with vehicle (=1.0). Error bars represent the mean ± s.d. (*n* = 3). Three independent experiments were performed for b and c and each experiment contain three biological replicates. The bars for b represent the mean ± s.d. of averaged values from each experiment. The bars in c are the mean ± s.d. of biological triplicates from a representative set.

The role of xCT in driving both glutathione production and the expression of glutathione-utilizing enzymes such as GPX4 suggests that its expression may promote survival under oxidative stress conditions for breast cancer cells that have elevation in xCT expression. We first identified two TNBC cell lines, MDAMB231 and CAL-120, as having increased expression of both SLC7A11 and SLC3A2, while two commonly used non-transformed mammary epithelial lines, MCF10A and MCF12A, were found to express only SLC3A2 but not SLC7A11, and are thus xCT-negative (Figure 3a,b). Next, we found that the breast cancer lines were highly resistant to death induced by hydrogen peroxide (Figure 3c). This suggests that xCT-positive breast cancer cells have a selective advantage against oxidative stress.

### 3.4. TNBC Cells Are Paradoxically Hypersensitive to Targeting of Anti-Ferroptotic Machinery by Erastin and Rsl-3

Our results thus far suggest that xCT positive breast cancer cells—Those expressing high levels of both SLC7A11 and SLC3A2—Are able to drive GPX4 expression through the selenocysteine biosynthesis pathway, and also are able to resist lipid prooxidant-induced death. This is in line with the known function of both xCT and GPX4 in protecting against ferroptosis in various cell stress states.

**Figure 4:**
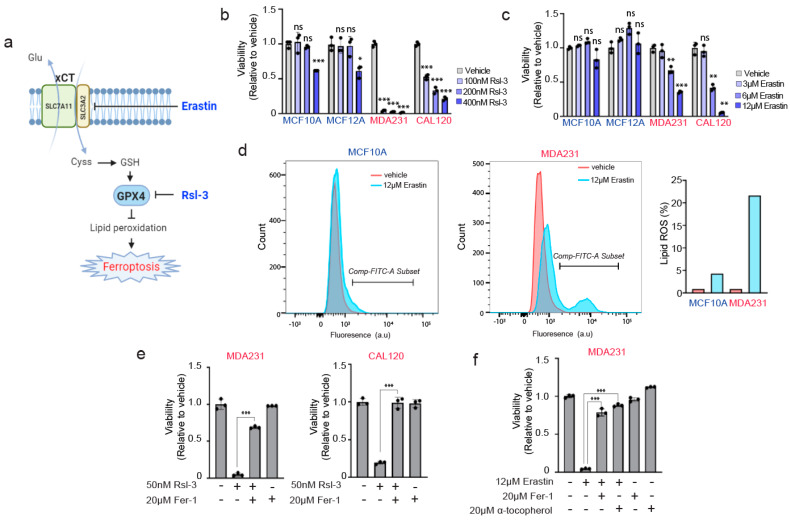
xCT/GPX4 suppression of ferroptosis is required in transformed, xCT-positive breast cancer cells but not in non-transformed breast cells. (**a**) Schematic diagram of a known working mechanism of Erastin and Rsl-3. Created with Biorender (https://app.biorender.com/) (**b**,**c**) The relative viabilities of Rsl-3 (**b**) and Erastin (**c**) treated non-transformed breast and breast cancer lines. The viabilities were measured at 24 h and 72 h after treatment of Rsl-3 and Erastin, respectively; values are relative to vehicle-treated control for each cell line (=1.0) (**d**) FACS analysis of Bodipy 581/591 C11 lipid oxidation in vehicle and Erastin treated MCF10A and MDAMB231 cells. The Bodipy staining was performed at 72 h after Erastin treatment. Red and Blue indicate vehicle and Erastin treatment conditions, respectively. Comp-FITC-A subset was defined as a lipid ROS positive cells, which is indicated in the right bar graph. (**e**) The viabilities of breast cancer lines treated with Rsl-3 and/or the lipophilic antioxidant/ferroptosis inhibitor ferrostatin-1 (Fer-1). The viabilities were measured at 24 h after treatment of the indicated compounds. (**f**) The viabilities of MDAMB231 treated with Erastin and/or lipophilic antioxidants (ferrostatin 1, α-tocopherol). The viabilities were measured at 72 h. Three independent experiments were performed for (**b**,**c**,**e**,**f**) and each experiment contain three biological replicates. The bars for (**b**,**c**,**e**,**f**) are the mean ± s.d. of biological triplicates from a representative set. Data marked with a one (*), two (**) or three (***) asterisks indicate *p* values of <0.05, <0.01 and <0.001. ns: no significance.

We next examined the sensitivity of these cancer and noncancer cells to prolonged exposure to agents targeting the xCT and GPX4 anti-ferroptotic machinery, Erastin and Rsl-3 (Figure 4a). Surprisingly, in contrast to their increased resistance against reactive oxygen species, we found that MDAMB231 and CAL120 cells were hypersensitive to both Erastin and Rsl-3 relative to the nontransformed lines. We observed that treatment of Erastin, even in the absence of a prooxidant insult such as hydrogen peroxide, induced a dramatic loss of cell viability (Figure 4b,c) and significant accumulation of lipid peroxidation species (Figure 4d). The loss of viability induced by Erastin or Rsl-3 treatment appeared to be caused by lipid peroxidation and ferroptosis, as they were rescued by the lipid antioxidant/ferroptosis inhibitor ferrostatin-1 or α-tocopherol (Figure 4e,f). Thus, while the TNBC cells had an augmented defense system mediated by xCT and GPX4 against oxidative stress (Figure 3c), they were paradoxically more dependent on these pathways, compared to normal cells, to prevent ferroptosis. This suggests an “addiction scenario” that can be further explored in future studies for optimal therapeutic targeting in breast cancer.

## 4. Discussion

The expression of both SLC7A11 and SLC3A2 subunits can be a marker for xCT function and selenoprotein production capacity of breast cancer cells. xCT consists of two subunits, SLC7A11 and SLC3A2. High expression of either SLC7A11 and SLC3A2 has been previously reported in several tumor types. SLC7A11 is highly expressed in many types of tumors such as acute myeloid leukemia, breast cancer, colorectal cancer, hepatocellular carcinoma, glioma, etc., and its high expression is associated with poor prognosis of patients with cancers [13,14]. SLC3A2 is highly expressed in breast cancer [15] and osteosarcoma [16]. Especially, the expression of SLC7A11 alone has been utilized to evaluate the expression of xCT [17,18,19,20,21,22]. To our knowledge, there has not been an approach to determine the expression of both SLC7A11 and SLC3A2 to evaluate the prognostic value of xCT. However, we previously showed that depletion of any subunits makes xCT nonfunctional [4], which means that confirming the expression of one subunit may not represent the existence of functional xCT in cells. In addition, SLC3A2 also comprises the heavy subunit of the large neutral amino acid transporter (LAT1) with a light subunit protein encoded by the SLC7A5 gene [23] which indicates that SLC3A2 may be expressed as a part of LAT1 complex in some cases. Thus, evaluating one subunit could not be an accurate marker for functional xCT. In this study, the expression of both subunits was evaluated side-by-side to evaluate a level of potentially functional xCT in breast normal and cancer tissues. Some tumors expressed only one subunit and others expressed both subunits which we defined as a xCT high tumor. The significantly elevated expression of GPX4 in xCT-positive tumors versus their paired normal tissues, or versus xCT-negative tumors, supports the notion that tumors expressing high levels of both subunits could be an indicator for the existence of the functional xCT which leads to high selenium uptake and upregulation of selenoproteins. However, this relationship between expression levels of xCT subunits and selenoproteins should be validated with larger sets of paired samples to further clarify the effect of the functional xCT on selenium uptake and its relationship with the expression of various selenoproteins in tumor pathophysiology.

Erastin and xCT inhibitors as a strategy to inhibit selenium uptake and GPX4 production in cancer cells. Erastin is currently being explored as a cancer therapy agent, in large part due to its role in inducing ferroptosis. Sulfasalazine and sorafenib are other compounds that have been similarly explored as xCT inhibitors, although they may have wider effects and target profile compared to Erastin [24,25]. Modified compounds based on Erastin with improved stability and/or bioavailability have also been developed [26]. Therefore, fully understanding the mechanism for Erastin and related compounds is of importance in further developing these as a therapeutic strategy. Here we show that Erastin drastically impairs extracellular thiol reduction, selenium uptake, and expression of the selenoprotein GPX4. This finding is significant, as the current model of Erastin mode of action only considers that of impairing glutathione biosynthesis. According to our findings, impaired production of GPX4, a key enzyme implicated in ferroptosis prevention which uses glutathione as substrate, is an additional important mechanism of action for Erastin/xCT inhibitors, one that by itself could have ferroptosis triggering properties even if glutathione production were to somehow be restored. A potential workaround for cancer cells around impaired GPX4 production is that aside from the common dietary selenium compound selenite, there are other independent routes by which selenium could be obtained, in particular recycling of selenocysteine obtained from selenium carriers such as SEPP1, or from selenomethionine, both of which would involve the action of selenocysteine lyase (SCLY) in forming selenide from selenocysteine, prior to its conversion to selenocysteinyl-tRNA. Thus, impairing this route of selenocysteinyl-tRNA formation in conjunction with xCT inhibition could have even more potent effects, and the potential functions of SCLY in cancer and ferroptosis should be explored in future studies.

‘Addiction’ of breast cancer cells to xCT/GPX4 anti-ferroptotic machinery. Conversely to the known effect of xCT inhibitors such as Erastin sensitizing or triggering ferroptosis, the increased expression or activity of xCT is implicated in the increased resistance of cancer cells against insults that can trigger ferroptosis, such as hypoxia or chemotherapy [27,28,29]. Along similar lines, the role of selenium in producing antioxidant selenoproteins forms the very basis of selenium being considered an antioxidant nutritional supplement [30,31]. Recently, selenocysteine delivered via synthetic peptide was shown to protect against ferroptosis in a stroke model [32]. We also recently showed that CRISPR disruption of selenocysteine biosynthesis machinery (SEPSECS, PSTK) hypersensitizes cancer cells against the lipid prooxidant tert-butyl hydroxide (TBH) [4]. Therefore, it was not surprising that the breast cancer lines MDAMB231 and CAL120, which have elevated expression of both xCT subunits, have increased resistance against hydrogen peroxide compared to the nontransformed immortalized lines MCF10A and MCF12A, which are xCT-negative (Figure 3). What was highly unexpected was that at the same time, MDAMB231 and CAL120 are hypersensitive to the drugs which directly target the machinery that allowed them to resist hydrogen peroxide, namely xCT (Erastin) and GPX4 (Rsl-3). This suggests a scenario in which anti-ferroptotic machinery is upregulated in these cancer cells but at the same time they are ‘addicted’ or highly dependent on this machinery even in the absence of an insult such as hydrogen peroxide. 

How are why are cancer lines addicted? One possibility involves selenide, which is formed by xCT activity in the process of selenocysteine biosynthesis (which ultimately results in GPX4 production). We have shown that the accumulation of hydrogen selenide gas formed during selenocysteine biosynthesis is toxic to cancer cells by increasing ROS levels [4]. As xCT-positive cancer cell lines are producing more selenide, they may be more dependent on the end product GPX4 to neutralize selenide-induced oxidative stress. As a second possibility, cancer cells might be more detrimental to GPX4 inhibition because they have a higher level of polyunsaturated fatty acids (PUFAs). Basal-like breast cancer cell lines are susceptible to ferroptosis due to the expression of acyl-CoA synthetase long-chain family member 4 (ACSL4) which enriches cellular membranes with long polyunsaturated fatty acids (PUFAs) [33]. Since ACSL4 is expressed higher in breast cancer compared to the adjacent normal tissue [34], and PUFAs are targets of lipid peroxidation, cancer cells might be more susceptible to lipid peroxidation and ferroptosis, thus GPX4 is more essential in cancer cells. As a third possibility, as the xCT-positive cancer cells are expected to have increased glutathione production and GPX4 expression, the resulting increased antioxidant capacity may be accompanied by a reduced selective requirement for other anti-ferroptotic or prosurvival signaling activities, meaning that when this xCT/GPX4-mediated protection is removed, these cancer cells are vulnerable compared to ‘nonaddicted’ cells.

## 5. Therapeutic Implications and Concluding Thoughts

We have shown that cancer cells that overexpress both SLC7A11 and SLC3A2 are the ones that should be considered xCT-positive, and that this pathway, through the selenocysteine biosynthesis pathway mediated production of GPX4, impacts a cancer cell’s sensitivity to various ferroptotic inducers. While future studies should delineate the exact underlying mechanism, our finding that xCT positive cancer cells are simultaneously resistant to lipid peroxidation insult yet hypersensitive to xCT or GPX4 inhibitors raises important therapeutic implications. There are two classes of ferroptotis inducers: those which directly trigger peroxidation such as hydrogen peroxide, and those that target a cell’s defense against lipid peroxidation. Our results imply that in xCT positive cancers, the latter is the desirable approach. xCT is widely reported to be upregulated in different subtypes of cancers, and has been linked with oncogenic mutations such as Keap1 loss, and associated with clinical parameters such as chemoresistance. Our findings provide a starting point and rationale for targeting anti-ferroptotic machinery of cancer cells depending on their xCT status, which can be further developed in future studies.

## Data Availability

All raw data that support the findings of this study are available from the corresponding author upon request. Breast cancer datamining was carried out using GEPIA web tool which utilizes datasets from The Cancer Genome Atlas and GTEx datasets (http://gepia2.cancer-pku.cn/).

## Supplementary Materials

The following are available online at https://www.mdpi.com/2076-3921/10/2/317/s1, Supplementary Figure S1. Expression profiles of SLC7A11 (a) and SLC3A2 (b) in total 33 types of normal and tumor tissue samples. Supplementary Figure S2. Prognostic values of SLC3A2 and SLC7A11 in patients with breast cancer. Supplementary Figure S3. Cell viability is not affected by short-term treatment of Erastin. Supplementary Figure S4. Uncropped image of blots. Supplementary Table S1. A list of materials used in this study. Supplementary Table S2. Information of cell lines and media used in this study.
