# Supplementary figures and images for: xCT-Driven Expression of GPX4 Determines Sensitivity of Breast Cancer Cells to Ferroptosis Inducers
Source: Antioxidants (Basel). 2021 Feb 20;10(2):317. doi: 10.3390/antiox10020317 (PMC7923775; doi:10.3390/antiox10020317)

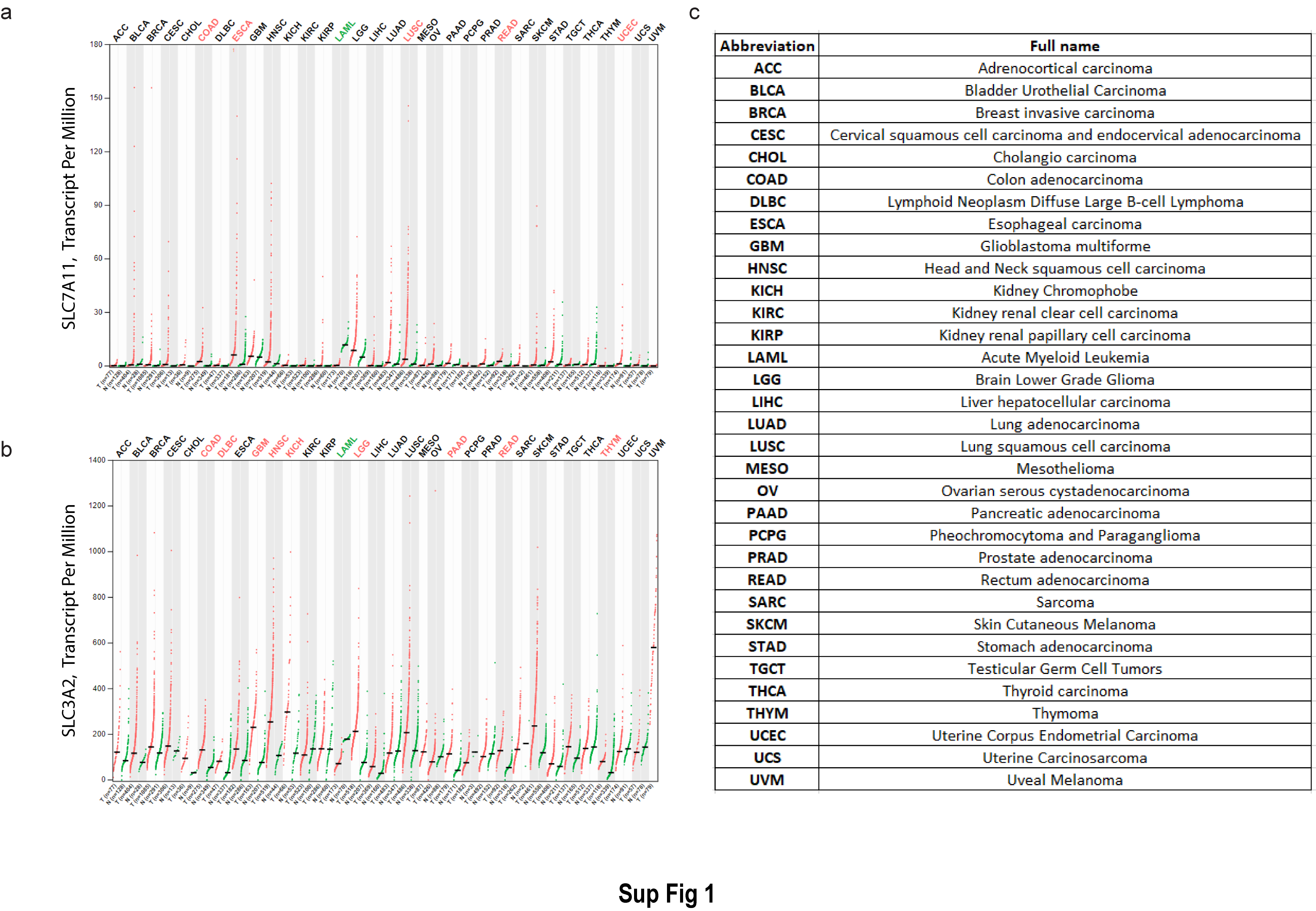

Supplement: Supplementary file 1 [file antioxidants-10-00317-s001.zip › Sup Fig 1.tif]

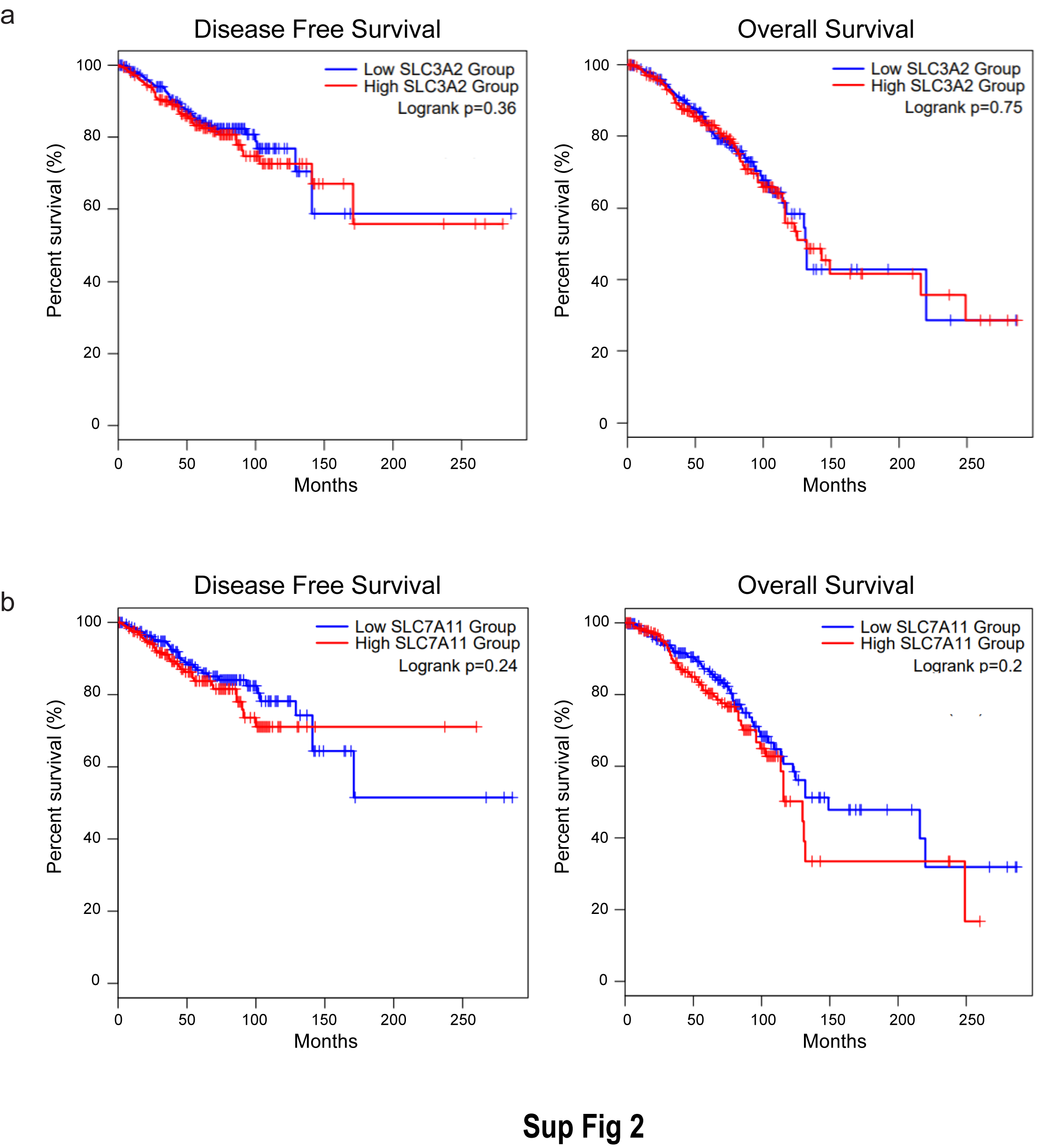

Supplement: Supplementary file 1 [file antioxidants-10-00317-s001.zip › Sup Fig 2.tif]

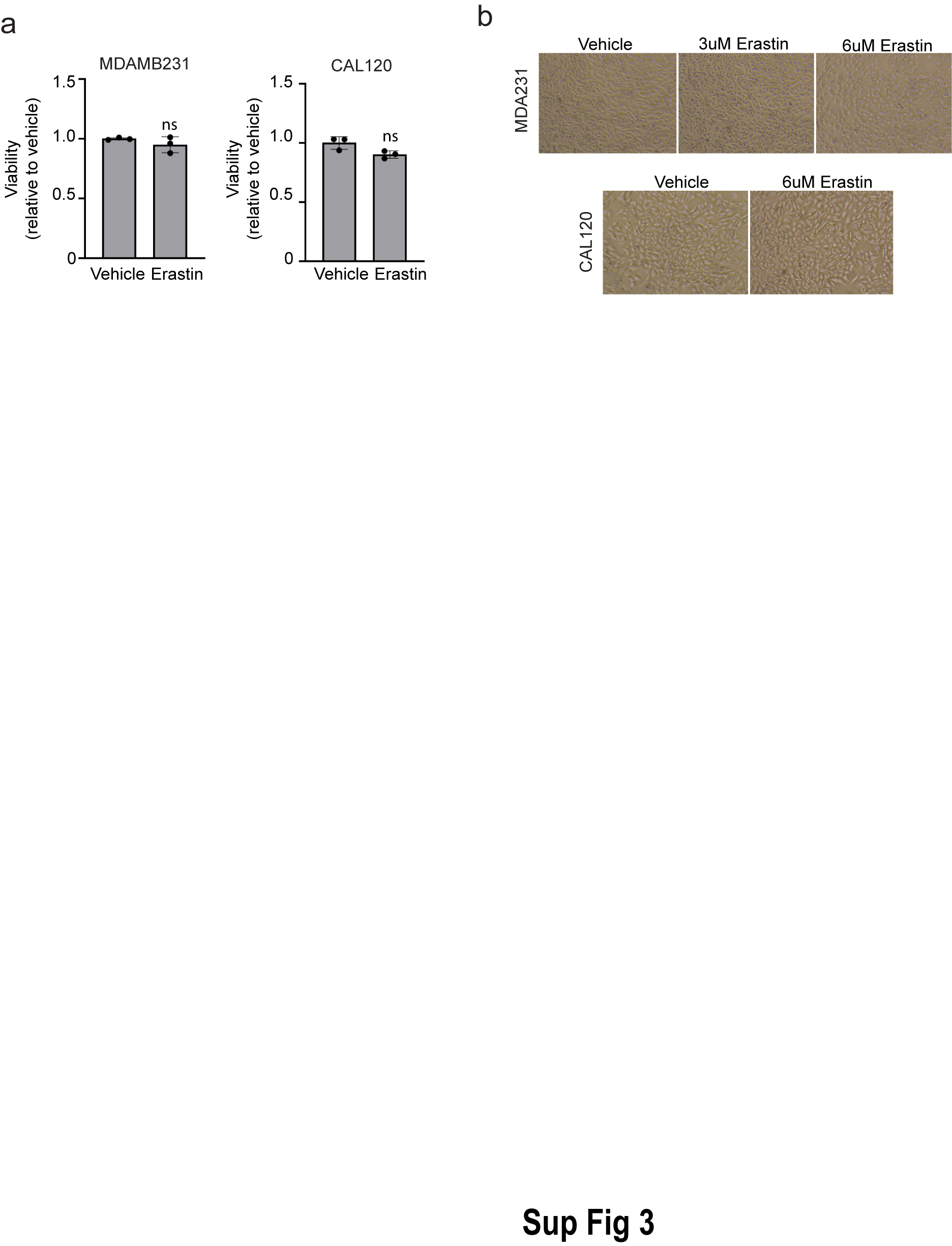

Supplement: Supplementary file 1 [file antioxidants-10-00317-s001.zip › Sup Fig 3.tif]

**b**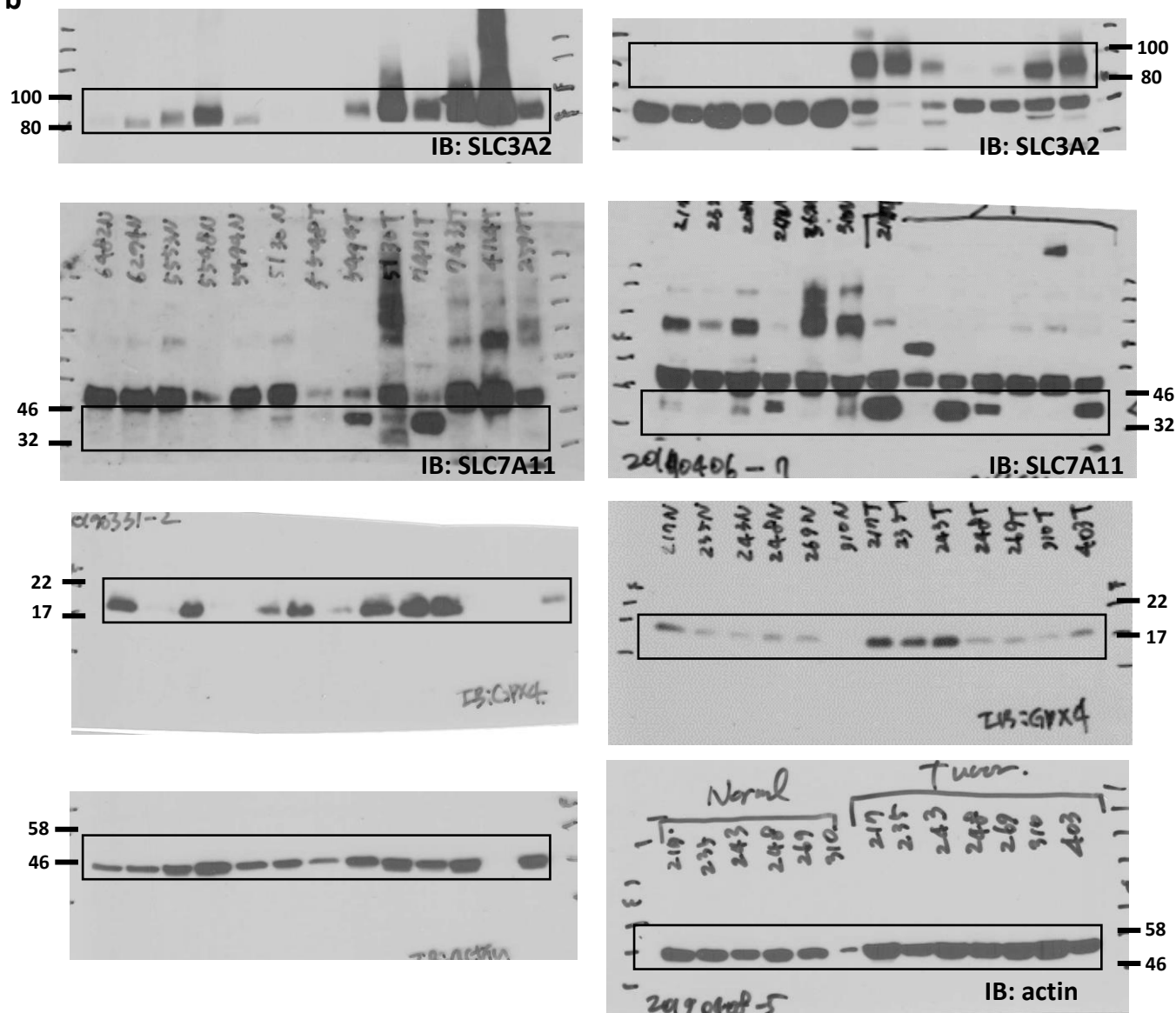**Fig 1**

d

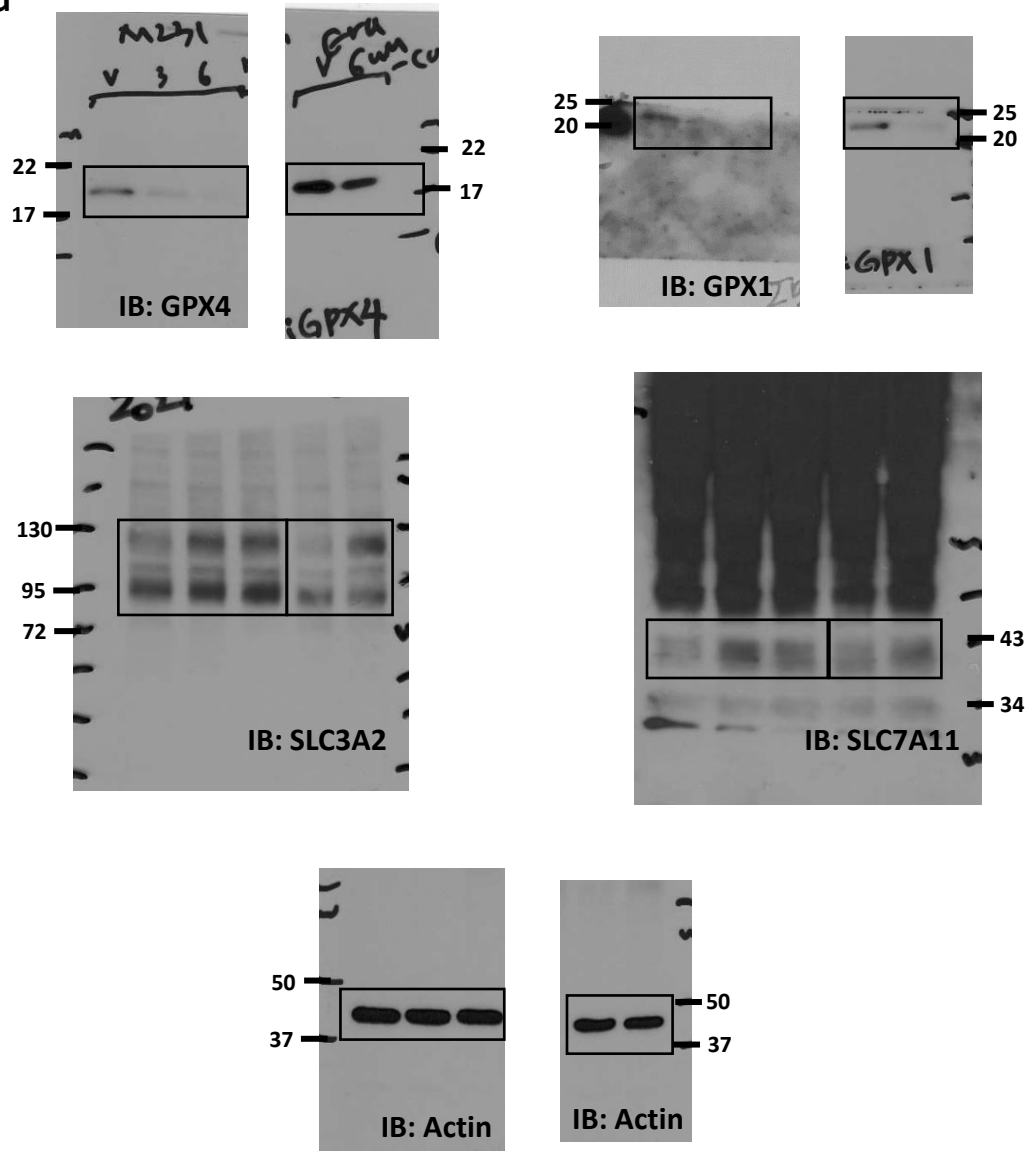

Fig 2

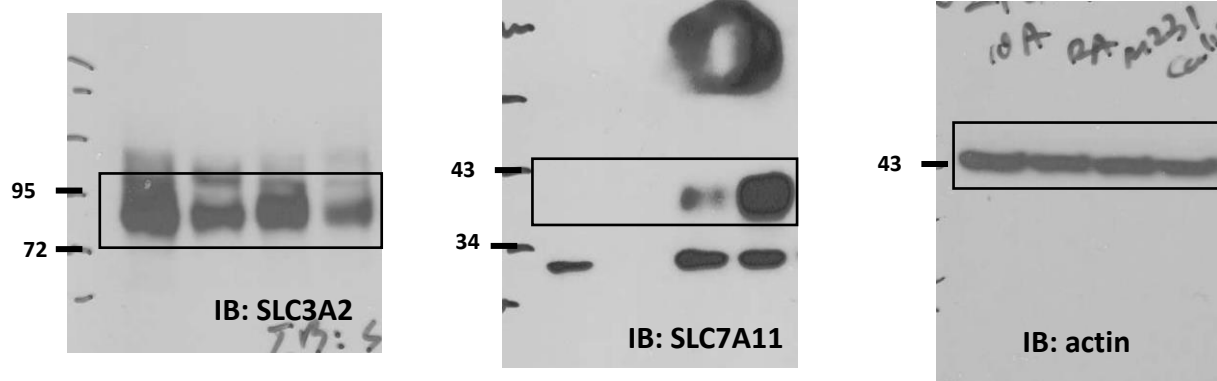

**Fig 3**

Supplement: Supplementary file 1 [file antioxidants-10-00317-s001.zip › Sup Fig 4.pdf]
